# Addition and Subtraction Theory of TCM Using Xiao-Chaihu-Decoction and Naturopathy in Predicting Survival Outcomes of Primary Liver Cancer Patients: A Prospective Cohort Study

**DOI:** 10.1155/2016/4723530

**Published:** 2016-10-24

**Authors:** Min Dai, Yue-Wu Yang, Wen-Hai Guo, Feng-Lin Wang, Ge-Min Xiao, Yang-Mei Li, Hong-Zhi Yang

**Affiliations:** Department of Traditional Chinese Medicine, The Third Affiliated Hospital of Sun Yat-sen University, Guangzhou 510630, China

## Abstract

To investigate the therapeutic effect of combined Xiao-Chaihu-Decoction and naturopathic medicine therapy on survival outcomes of patients' PLC. In XCHD group (*n* = 76), patients were treated with Xiao-Chaihu-Decoction in accordance with the addition and subtraction theory of TCM; in NM group (*n* = 89), patients were managed by naturopathic medicine; in combined group (*n* = 70), the same volume of Xiao-Chaihu-Decoction combined with naturopathic medicine procedures was applied. There were no evident statistical differences of age, gender, KPS score, body weight, smoking status, AFP levels, HbsAg status, TBIL levels, tumor diameters, and numbers among different groups, showing comparability among groups. No significant difference was found regarding the total remission rate and stability rate of tumors in patients treated by Xiao-Chaihu-Decoction and naturopathic medicine, except the combined therapy. KPS scores were significantly improved after treatment among groups. After treatment, 52.8% cases maintained a stable or slight increase in weight, of which 42.1%, 48.3%, and 70.0% cases maintained weight stably in the XCHD group, NM group, and combined treatment group, respectively. Xiao-Chaihu-Decoction associated with naturopathy may predict improved prognostic outcomes in PLC patients, along with improved remission and stability rates, increased KPS scores, and stable weight maintenance.

## 1. Introduction

Primary liver cancer (PLC) is the sixth most common malignancy worldwide, which includes two major types, hepatocellular carcinoma (HCC) and intrahepatic cholangiocarcinoma (ICC) [1]. The prevalence of both types of PLC has remained to be highly increased in both Western and Eastern countries [2]. Estimated data regarding the mortality of PLC shows an upward trends particularly in middle to senior people, and there is an obvious more favorable trend in women than in men [3]. Besides, the gender distribution of HCC and ICC is also different, with a much greater male preponderance for HCC than for ICC [2]. Inducement factors of HCC and ICC are at least in part different; excessive alcohol consumption, cirrhosis, and diabetes are all involved in being partially responsible for the incidence and development of both malignancies [4, 5]. In addition, major risk factors for HCC are suggested to be chronic infections with hepatitis C virus (HCV) and hepatitis B virus (HBV) [6]. However, documented evidence in both the HCV- and HBV-related HCC does not fully explain increased incidence rates in PLC since 1/5 to 1/2 of the HCC cases remain idiopathic [7]. Meanwhile, previous history of biliary tract or liver diseases and inflammatory bowel disease may also be partially correlated with the development of ICC [8]. Yet these factors only account for a relatively small part of the attributable risk factors of this disease [9]. Importantly, the incidence of PLC in China is still the highest around the world, even though a declining trend of PLC morbidity and mortality has been observed in several parts of China, due to at least in part the improvement in the pathological confirmation and target treatment strategies of this disease [10].

Surgical resection is still the predominant choice for most clinicians aiming at achieving removal of tumors completely [11]. However, surgical treatment cannot entirely relieve the current urgent status, mostly due to the reason that this type of cancer cannot be timely diagnosed most frequently, and it is characterized by postoperative aggressive lymph nodes metastasis and high tumor recurrence rate [12]. With respect to the above situation, multidisciplinary therapy is therefore promoted for the treatment of PLC in recent decades.

Herbal medicines and herbs have been strongly correlated with peoples' daily life especially in China and some Southeast Asian countries. Plant elements and extracts are the most commonly used substance by far and also involves animal and mineral products, through decoction, pills, ointment, granule, and tablet to produce specific pesticide effects based on different patients' states for the benefits of disease healing [13, 14]. Being widely recognized as antineoplastic medicine for a long time, Chinese herbal medicine has been adopted in the treatment infectious diseases, hypertension, and cardiovascular diseases [15–17]. More and more traditional Chinese medicine (TCM) has been used for treatment of PLC in China with the use of herbs or herbal formulae [18]. TCM is particularly preferred for elderly patients or those with advanced disease, exerting variable effects such as inhibition of tumor angiogenesis, induction of cancer cell apoptosis, regulation of immunity, and analgesia [19]. Furthermore, naturopathic medicine, as a form of alternative medicine employing a wide array of natural modalities, favors a holistic approach with noninvasive procedures and avoids the selection of surgical approach or Western medicine treatment [20]. This may include herbalism, acupuncture, massage therapy, and nature cures (diet/nutrition, exposures to nature elements, fasting, etc.) and physical medicine (sports medicine, exercise, etc.) [21] and contribute to restoring human body's innate ability to heal itself without the adverse effects of conventional medicine. However with features of unnecessarily safer or ineffective than other surgical or chemical treatments, any treatment capable of eliciting an effect may also have deleterious side effects.

In the study, a hypothesis was promoted that Xiao-Chaihu-Decoction combined with naturopathic medicine therapy may have efficacious synergistic reaction for PLC. Few previous studies were found focused on the clinical experiment of this topic. A retrospective investigation was thereof conducted to investigate the efficacy of TCM treatment combined with radical surgery on survival and recurrence outcomes in PLC patients.

## 2. Methods

### 2.1. Ethics Statement

This research procedure was conducted conforming to the ethical guidelines established by the ethics committee at the Third Affiliated Hospital of Sun Yat-sen University, Guangzhou, China. In accordance with the Declaration of Helsinki 1964, the written informed consent for relevant information gathering (general data, primary symptoms, concomitant symptom, pulse condition, signs, etc.) and treatment details (diagnosis and treatment of TCM and Western Medicine, TCM syndromes diagnosis during hospitalization, symptomatic transformation, effectiveness, etc.) was signed and obtained from all subjects enrolled in the study or their guardians prior to the performance of predefined treatment strategies.

### 2.2. Patient Eligibility

The present prospective cohort study was conducted to evaluate the efficacy of TCM treatment in the incorporated eligible patients. From June, 2012, to January, 2014, a total of 235 PLC outpatients who visited our hospital were randomly enrolled in this study. Included patients were recorded to have a previous history of surgical resection. There were 195 males and 40 females with different ages ranging from 20 to 75 years, with a mean age of (58.34 ± 5.26 years). Both pathological diagnosis and cytological examinations (biopsy or surgical resection specimens) were performed to confirm PLC in association with Standard Specification for diagnosis and treatment of PLC (version 2011) [22]. Inclusion criteria of eligible patients were listed as follows: (1) patients who were histologically confirmed with PLC by pathological or cytological examination; (2) patients who had previous surgical resection within the last six months; (3) patients who had no other tumors or distant metastasis preoperatively and intraoperatively; (4) in accordance to the new American Joint Committee on Cancer (AJCC)/International Union Against Cancer (UICC) staging systems, eligible subjects who were incorporated with a clinical stages II~III [23]; (5) patients who were under the Child-Pugh classification status of A by Child-Pugh Score of the liver function [24]; (6) patients who had no absolute contraindications to liver resection; (7) patients who received no previous Chinese herbal medicine treatment within the last 3 months; (8) patients who had no previous history of internal diseases; (9) patients who were capable and willing to accept the program for treatment and had complete clinical and follow-up data.

Exclusion criteria were as follows: (1) patient diagnosed with secondary hepatocarcinoma; (2) patients who also had other malignant tumors; (3) patients who had other serious underlying diseases, such as the respiratory system related diseases, cardiovascular and cerebrovascular diseases, or mental disorder; (4) patients who received previous anticancer therapy, for instance, chemotherapy or radiotherapy, during the latest 3 months and got effects (comparison regarding tumor size and tumor related indexes, etc., before and after treatment indicated positive effect, and patients showed no relapse); (5) patients with refractory ascites or hepatic encephalopathy; (6) patients who had previous history of esophageal varices bleeding rupture; (7) patients with metastatic tumor of liver promoted by CT/MRI scanning; (8) women who were in their pregnancy and breastfeeding period; (9) patients without complete clinical information or unwilling to undergo this procedure, or patients with incomplete follow-up data.

Included 235 patients were divided into the following three groups according to different treatment approaches that patients received based on their physical status: (1) there were 76 patients (61 males and 15 females, 24–72 years old, mean age of 51.05 ± 13.63 years) treated with the addition and subtraction theory of TCM using Xiao-Chaihu-Decoction, named as the XCHD group. Further, another 89 patients (76 males and 13 females, 23–75 years old, mean age of 50.10 ± 17.04 years) were treated with naturopathic medicine therapy, called the NM group. Remaining 70 cases (58 males and 12 females, 25–68 years old, mean age of 49.33 ± 12.41 years) were divided into the combined group and treated with the same volume of Xiao-Chaihu-Decoction combined with naturopathic medicine therapy. Clinical comparison and analysis among groups were conducted subsequently.

### 2.3. Treatment Regimens in the XCHD Group

Patients in the XCHD group received Xiao-Chaihu-Decoction regimen, supplemented with salvia miltiorrhiza,* Panax notoginseng*, peach kernel, and angelica sinensis for the activation of blood circulation to dissipate blood stasis, detumescence, and odynolysis. In addition,* Arisaema* cum Bile was added to eliminate phlegm;* Lobelia chinensis* Lour, and* Taxus chinensis* were also added for the enhancement of antitumor immunity. In addition, with regard to the addition and subtraction theory of TCM, there were six different situations that were planned to treat with different herbals: (1) for patients with the symptom of deficiency of fluid due to dryness and heat,* Dendrobium* and habitat were added; (2) for patients with the symptom of deficiency of stomach-yin,* Adenophora*,* Polygonatum odoratum*, and Ophiopogon were added; (3) for patients with kidney yang deficiency,* Tetradium ruticarpum*,* Cinnamomum cassia* Presl, and Fuzi were used; (4) Frankincense and Myrrh were selected to alleviate the pain; (5) for patients with the symptoms of night urination and back pain, prepared Radix Rehmanniae and Fructus corni were comprised; (6) for hyperhidrosis patients,* Astragalus membranaceus*, radices ephedrae, light wheat, Fructus Tritici Levis, and radix oryzae glutinosae were added.

### 2.4. Treatment Regimens in the NM Group

The following naturopathic treatment was used in the NM group, including acupoint application, nature cures (diet/nutrition), combined therapeutic apparatus, and meridian therapy. Participants in the NM group were informed to receive all the above-mentioned therapies at the same time; besides, combined therapeutic apparatus was applied in patients for the first month, as then subjects were asked to receive meridian therapy for the following one month. Acupoint application therapy, which is based on the theory of meridians and collaterals in traditional Chinese medicine, to grind the drug into fine powder, tune into paste state, or into ointment, pill or cake agent, or boiled paste, and so forth, and then apply directly on the acupoint or the affected area, is a noninvasive pain acupoint therapy with the usage of traditional Chinese herbal medicine. Detailed acupoint application was based on patients' conditions; different treatment regimens were applied in different patients according to different syndrome differentiation types, once every day, all for ten times as one course of treatment, in a total of three courses.

#### 2.4.1. Acupoint Application

Detailed acupoint application was listed as follows based on patients' conditions: (1) for damp-heat constitution: Talc, 45 g; skullcap, 30 g; capillary wormwood, 30 g; Rhizoma Acori Tatarinowii, 18 g; through grass, 15 g; wrinkled giant hyssop, 12 g; forsythia, 12 g; amomum cardamomum, 12 g; Mentha haplocalyx, 12 g; Rhizoma Belamcandae, 12 g; (2) for phlegm-dampness constitution: wrinkled giant hyssop, 12 g;* Magnolia officinalis*, 12 g; Rhizoma Pinelliae Preparata, 12 g; fuling, 15 g; Almond, 9 g; semen coicis, 15 g; amomum cardamomum, 9 g; grifola, 15 g; rhizoma alismatis, 12 g; pericarpium citri reticulatae, 9 g; Chinese atractylodes, 9 g; (3) for yang-deficiency constitution: radix bupleuri, 9 g;* Pinellia ternata*, 12 g; skullcap, 9 g; dangshen, 6 g; Baked Licorice, 9 g; Fructus Ziziphi Jujubae, 9 g; ginger, 15 g; (4) for qi-insufficiency constitution:* Astragalus mongholicus*, 30 g; rhizoma anemarrhenae, 9 g; radix bupleuri, 6 g; Platycodon grandiflorum, 6 g;* Cimicifuga*, 6 g; Baked Licorice, 24 g; (5) for Chlic distention (obstruction of the circulation of vital energy in the gallbladder) and minor yang constitution with excess heat, Da-Chaihu-Decoction was used (radix bupleuri, 12 g; skullcap, 9 g;* Paeonia lactiflora* Pall, 9 g; Rhizoma Pinelliae Preparata 9 g; ginger, 15 g; fructus aurantii immaturus, 9 g; Fructus Ziziphi Jujubae, 9 g; rheum officinale, 6 g); (6) for haemostasis constitution: rheum officinale, 9 g; angelica, 9 g; Radix Rehmanniae Recens, 15 g; peach kernel, 12 g; safflower, 9 g; fructus aurantii, 12 g; Radix Paeoniae, 15 g; radix bupleuri, 9 g; licorice, 9 g;* Platycodon grandiflorum*, 12 g;* Ligusticum wallichii*, 12 g; radix cyathulae, 12 g; Radix Dipsaci, 9 g; horny spine, 9 g; (7) for kidney yang asthenia constitution: prepared Radix Rehmanniae, 15 g; Chinese yam, 15 g; Fructus corni, 15 g; fuling, 15 g; bark of tree peony root, 15 g; rhizoma alismatis, 15 g; cinnamon, 3 g; monkshood, 3 g; (8) for yin deficiency constitution: radix glehniae, 15 g; Radix Ophiopogonis, 15 g; angelica, 12 g; Radix Rehmanniae Recens, 30 g; the fruit of Chinese wolfberry, 15 g;* Melia toosendan*, 15 g. The above-mentioned medicine were milled into powder, mixed with electrolytic water evenly, and stored in a refrigerator at 4°C for the following acupoint application process.

Relevant moxibustion points were selected based on the theory of meridians and collaterals in traditional Chinese medicine: (1) for damp-heat constitution: Ganshu (bilateral), Tanshu, Pishu, He-Sea point, and Sanyinjiao; (2) for phlegm-dampness constitution: Danzhong, Chungwan, Shuidao, Guiai, Rich and Prosperous, and Pishu; (3) for yang-deficiency constitution: Ganshu, Tanshu (bilateral), Qimen, Riyue, and Zhangmen; (4) for qi-insufficiency constitution: Qihai and Guanyuan, Shenshu (bilateral), Mingmen and Yaoyangkuan, Feishu, and Hsinshu; (5) for stagnation of qi physical: Ganshu (bilateral), Tanshu, Qimen, Riyue, and Zhangmen; (6) for haemostasis constitution: Xuehai (bilateral), Zusanli, Quchi, Pishu, and Ashi point (emmeniopathy: Zigong); (7) for kidney yang asthenia constitution: Hsiawan and Shenque, Qihai and Guanyuan, Shenshu (bilateral), Mingmen and Yaoyangkuan, and Guanyuanshu; (8) for yin deficiency constitution: Shuidao, Guiai, He-Sea point, Sanyinjiao, Taixi (bilateral), and Ashi point.

*Nature Cures (Diet/Nutrition)*. Patients in this group were also treated with nutrition and dietary changes: (1) to prevent cancer and to improve the resistibility of human bodies: vegetables [carrot root (1/2), tomato (1), and papaya (1/4)]; fruits [Tomatoes (4), apple (1/2), grapes (15), orange (1/2), and lemon (1)]; mixed ingredients [alkaline water (2.5 cups), dried seaweed (1/4 tablets), and black sesame (2 tablespoons)]; (2) for cancer prevention and anticancer: vegetables [beetroot (1/2), bean sprouts (1/2 cup),* Flammulina* volutes, carrot root (1), red cabbage (1/8), balsam pear (1/2), and pumpkin (1/8)]; fruits [tomatoes (2), grapes (10), and lemon (1)]; mixed ingredients [alkaline water (2 cups), ginger (5 pieces), Brazil nuts (5 grains), flaxseed (2 teaspoons), bee pollen (2 teaspoons), basil (8 leaves), and Chinese wolfberry (3 teaspoons)]. All the vegetables, fruits, and ingredients were whipped into the juice for drink.

#### 2.4.2. Combined Therapeutic Apparatus

Combined therapeutic apparatus was applied for discomfort improvement such as hypochondriac pain, stomach pain, diarrhea, indigestion, chills, shortness of breath, ascites, and other symptoms. The treatment was performed 20–40 min each time, lasting for one course of treatment.

#### 2.4.3. Meridian Therapy

This therapy was performed under the high magnetic field; patients were informed to keep prone positions on the treatment couch and treated with the meridian therapy instrument (YuXuanGong, Guangdong, China); acid bilges feeling was appropriate in patients. The initial treatment time lasted for 20 min, followed by 40 min each time over the course of 1 month, thus dredging the meridian.

Besides, relevant moxibustion points were different according to the above-mentioned patients' constitution. Meanwhile, patients in this group were also treated with nutrition and dietary changes (a glass of fruit and vegetable juice per day and two kinds of fruit and vegetable juice were randomly selected daily) to prevent cancer and to improve the resistibility of human bodies and exerting cancer prevention and anticancer effects. In addition, combined therapeutic apparatus was applied for discomfort improvement such as hypochondriac pain, stomach pain, diarrhea, indigestion, chills, shortness of breath, ascites, and other symptoms. The treatment was performed 20–40 min each time, once every day, lasting for one course of treatment. Meridian therapy was also performed under the high magnetic field; patients were informed to keep prone positions on the treatment couch and treated with the meridian therapy instrument (YuXuanGong, Guangdong, China); acid bilges feeling (indicating good treatment response, which was also identical to the state of “De-Qi”/the arrival of Qi in TCM) was appropriate in patients. The initial treatment time lasted for 20 min, followed by 40 min each time over the course of 1 month (once a day), thus achieving the effect of dredging the meridian.

### 2.5. Follow-Up

The survival outcomes of the patients were evaluated at the end of the treatment by telephone follow-up. Follow-up lasted for one year; patients' survival times and course of disease outcome were recorded in detail among groups. Tumor size changes before and after treatment were categorized based on WHO evaluation standard of curative effect for solid tumor [25]: (1) complete remission: lesions disappeared without new occurrence, associated with normal detection of tumor markers for over a month; (2) partial remission, tumors disappeared with the reduced maximum diameter over 30.0% for over a month; (3) stable disease: tumors got some relief with the maximum diameter reduction between complete remission and partial remission; (4) progression disease: lesions of the patients appeared again after the disappearance of those symptoms, or with total lesions maximum diameter increased over 20.0%.

### 2.6. Data Collection and Statistical Analysis

Data were obtained from hospital medical records and pharmacy records. Collected data included the patient's name, gender, age, body weights, smoking, tumor location, tumor numbers, tumor diameters, Karnofsky performance score (KPS), total bilirubin (TBIL), alpha fetal protein (AFP), HBsAg, tumor diameters and numbers, and patients or their families telephone. Among them, the evaluation of tumor volume (tumor response), KPS score, and body weight was performed before and after treatment.

The data were analyzed with the SPSS18.0 software (SPSS Inc., Chicago, IL, USA). The measurement data was given in the form of mean ± standard deviation, and categorical data were expressed as a percentage. Comparison among groups was performed by applying the one-way analysis of variance (ANOVA) test and between-groups comparison was conducted by using *χ* ^2^ test. Kaplan-Meier analysis calculated the survival estimates combined with the Log-rank test. All tests were bilateral and *P* < 0.05 indicated a statistical significance.

## 3. Results

### 3.1. Patients and Disease Characteristics

A sum of 235 patients diagnosed with PLC and who had a previous history of surgical resection were enrolled in this experiment, which consisted of 195 males and 40 females with different ages ranging from 20 to 75 years, with a mean age of 50.18 ± 14.65 years. All patients were preset to receive different treatment regimens in strictly accordance with their physical condition. There were no evident statistical differences of age, gender, body weight, smoking status, AFP levels, HbsAg status, TBIL levels, tumor diameters, and numbers among different groups before treatment, indicating good comparability among groups (Table 1).

### 3.2. Survival Analysis

**Figure 1:**
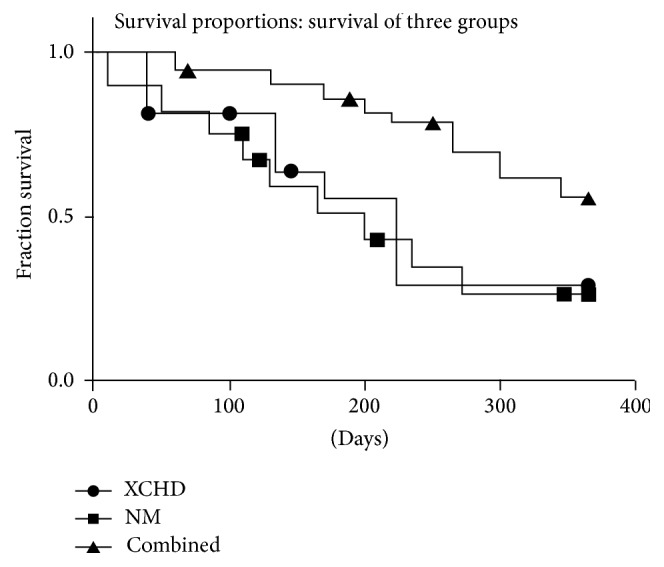
The Kaplan-Meier survival curve of three different treatment regimens for the treatment of primary liver cancer: (1) the addition and subtraction theory of TCM using Xiao-Chaihu-Decoction (XCHD group); (2) naturopathic medicine therapy (NM group); (3) Xiao-Chaihu-Decoction combined with naturopathic medicine therapy (combined group).

Through telephone follow-up, 156 cases died and 79 cases survived. Until January 2015, 10 cases were lost during the follow-up period, with a loss ratio of follow-up of 4.3%. Eligible patients were followed up for one year, and correspondingly the one-year survival rate was 33.6%, with a median survival period of 223 days. To be specific, the median survival period was 204 days, 193 days, and 295 days in the XCHD group, the NM group, and the combined group, respectively, with a one-year survival rate of 27.6%, 23.6%, and 52.7% correspondingly. The above results indicated that the one-year survival period was higher in the combined group than in the other two groups, associated with much higher one-year survival rate. The Kaplan-Meier survival curve was presented in Figure 1. As shown in the figure, overall survival in the Kaplan-Meier survival curve appeared significantly better in the combined group when compared to the XCHD group and the NM group; in addition, overall survival indicated to be slightly better in the NM group in the middle-term follow-up period but was further worse when compared to the XCHD group.

### 3.3. Tumor Responses, KPS Scores, and Body Weight Changes after Treatment

Tumor responses associated with different therapies were evaluated accordingly. In the included 235 patients, 152 patients were evaluated for tumor size. As presented in Table 2, the total remission rate was 40.1% in the evaluated patients, of which the tumor remission rate was 35.2%, 39.3%, and 47.6% in the XCHD group, the NM group, and the combined treatment, respectively. In addition, the total stability rate was 69.7%, and the stability rate of the combined therapy was 73.8%, higher than the other two groups, where the stability rate of XCHD and NM treatment was 68.5% and 67.9%, respectively.

In addition, improvement in the KPS scores was found among three groups. To be specific, KPS scores showed evident increased tendency before and after treatment in the XCHD group and the NM group (former: 65.21 ± 10.14 versus 78.11 ± 11.09; latter: 69.33 ± 11.47 versus 81.79 ± 11.93), showing statistical significance (both *P* < 0.05). KPS scores were also increased in the combined group after treatment, but no apparent statistical significance was found largely due to the generally higher scoring in this group (87.67 ± 12.62 versus 86.43, *P* > 0.05).

After treatment, 52.8% of cases maintained a stable or slight increase in weight, of which 42.1%, 48.3%, and 70.0% of cases maintained weight stability in the XCHD group, NM group, and combined treatment group, respectively. Among groups comparison results indicated that there was statistical difference with respect to the variation trend of weight, which was slightly decreased in both the XCHD and NM treatment groups after treatment in comparison to before treatment (former: 58.05 ± 9.43 versus 56.63 ± 9.03; latter: 60.16 ± 8.98 versus 58.00 ± 8.47); the differences were significant (both *P* < 0.05), whereas the body weight in the combined treatment group remained stable after treatment (*P* = 0.091) (Table 3).

## 4. Discussion

Most of threat and burden of liver cancer occur in developing countries and approximately half of the incidence and deaths were estimated to occur in China [26]. Efficacious therapies are therefore important for the prevention of various kinds of liver cancer. The present study was performed to identify the potential therapeutic roles of Xiao-Chaihu-Decoction combined with naturopathy in PLC patients who had a previous history of surgical resection. Corresponding results suggested that a combination of Xiao-Chaihu-Decoction and naturopathy may help a lot the improvement of prognostic outcomes in PLC patients.

With respect to Xiao-Chaihu-Decoction, a famous prescription in* Shanghanlun* [27] has been suggested to be workable for the treatment of breast cancer, type 2 diabetes, chronic glomerulonephritis, and so forth [28–30]; it has also been proved to be effective in the treatment of liver diseases because it can block the development of hepatitis to liver fibrosis and further to prevent the process of liver cancer through various pathways [31]. Related in vivo mechanisms include the following: inhibiting the replication of hepatitis virus, protecting liver cells, preventing liver damage, suppressing liver fibrosis, and immune regulation and antitumor effect [32–34]. For example, Xiao-Chaihu-Decoction may be involved in antihepatic fibrosis via inhibiting the activation of hepatic stellate cells, synthesis of collagen, and induction of correlated mRNA expression in the liver, further decreasing platelet-derived growth factor and growth factor B1, both of which are important factors for stimulating the formation of liver fibrosis and preventing liver fibrosis development to cirrhosis and eventually to liver cancer [35]. Most importantly, PLC is a disease that mainly correlated with HBV or HCV infections, Xiao-Chaihu-Decoction has obvious anti-HBV effect, and anti-HBV mechanism of Xiao-Chaihu-Decoction may be achieved by regulating the immune system [31, 36]. Besides, Xiao-Chaihu-Decoction may be responsible for the inhibition of the cell cycle G0/G1 stage of cancer cell line, as well as direct suppression of cell proliferation and apoptosis by human immune-deficiency virus infection, thereby inhibiting the transformation of liver cirrhosis to cancer, removing reactive oxygen species in a dose dependent manner, and suggesting an inhibitory effect in the initial stage of carcinogenesis [33, 37].

The essential role of naturopathy in the treatment of PLC was also proved in this study. In view of the belief in human body's ability to heal itself through a special vital energy or force guiding bodily processes internally without the adverse effects of conventional medicine, naturopathic practice is an increasingly significant part of the healthcare sector worldwide, having attracted more and more attention recently [38]. Naturopathic treatment has significant benefit especially in the management of patients who failed to be managed by curative treatment; it is also promoted to be another category of treatment in patients with end-stage malignancy [39–41]. Naturopaths often recommend exposure to naturally occurring substances, such as sunshine, herbs, and certain foods, as well as activities they describe as natural, such as exercise, meditation, and relaxation [42, 43].

In this study, we confirmed that there was certain improvement of survival outcomes in PLC patients, which was consistent with our previous speculation and in line with the above mechanism explanation. Acupoint application, diet/nutrition, combined therapeutic apparatus, and meridian therapy were performed in the NM group; all might be workable in exerting curative effects and improving patients' prognostic outcomes. Importantly, combined Xiao-Chaihu-Decoction with naturopathy was proved to contribute to a significant improvement in survival outcomes of PLC patients than single treatment regimen in this study. Possible reason for their synergistic treatment effect, though not validated in our study, was speculated to be correlated with the mechanism that the combination of Xiao-Chaihu-Decoction and naturopathy claimed a relatively stronger antitumor activity and associated with prolonged survival period in a great substantial portion of patients [39]. Besides, tumor response, KPS scores, and body weight changes after treatment also indicated better outcomes in those patients received combined treatment, which in turn confirmed that the association of Xiao-Chaihu-Decoction based on the addition and subtraction theory of TCM and naturopathy had significantly better curative effect in improving PLC patients' prognosis after surgical resection treatment.

Importantly, the main contributions of this paper are in designing the clinical effect of Xiao-Chaihu-Decoction and naturopathy in the treatment of PLC in view of the prognostic outcomes and other parameters such as tumor response, KPS, and weight. At present, there are few studies focused on the investigation of TCM treatment and naturopathy for PCL; besides, liver cancer is common worldwide with extremely high death rate annually. The promotion of the addition and subtraction theory of TCM using Xiao-Chaihu-Decoction and naturopathy may be helpful for providing new medical approaches for patients with liver cancer and also with other types of malignant tumors. Furthermore, there were tough selection criteria for eligible participants, and the application of different treatment strategies was in strict accordance with patients' constitution. As an observational study, patients' baseline characteristics were carefully compared to prove the comparability of included subjects; besides, experimental data were also recorded in detail among different groups, which in turn ensured the validity and comparability of the data. Nevertheless, there are some limitations of this study that should be taken into consideration. Firstly, as an observational study, the main limitation is the risk of the inherent deviation due to the confusion caused by the indications. There may be some differences in the final results compared to these concluded from randomized clinical trials, which should be more careful in the clinical decision-making process; randomized clinical trial is therefore planned to be performed to verify the above results in our future research. Secondly, there was a lack of exploration of confounding effects on the results; some variation existed in treatment approaches within the XCHD and NM treatment groups. For strengthening the reliability of results, further study should be involved to explore potential confounding effects that may affect results.

To sum up, the addition and subtraction theory of TCM using Xiao-Chaihu-Decoction and naturopathy may lead to improved prognostic outcomes of PLC, along with improved remission and stability rates, increased KPS scores, and stable weight maintenance. However, further investigation under a larger sample size study is needed to clarify in vivo mechanisms regarding how Xiao-Chaihu-Decoction and naturopathy combined exerting antitumor effect in the development of PLC and to provide further evidence of whether Xiao-Chaihu-Decoction may have therapeutic effect on the management of other human cancers or diseases, which can promote a medical and economic position of the TCM treatment in the management of liver disease and other human diseases without doubt.

## Figures and Tables

**Table 1:** Baseline characteristics among groups.

Group	XCHD group (*N* = 76)	NM group (*N* = 89)	Combined group (*N* = 70)	*P*		
				(XCHD group versus NM group)	(XCHD group versus combined group)	(NM group versus combined group)
Age (year)	51.05 ± 13.63	50.10 ± 17.04	49.33 ± 12.41	0.697	0.428	0.751
Gender (female/male)	61/15	76/13	58/12	0.382	0.687	0.663
KPS	65.21 ± 10.14	69.33 ± 11.47	87.67 ± 12.62	0.016	<0.001	<0.001
Body weight	58.05 ± 9.43	60.16 ± 8.98	59.73 ± 9.11	0.144	0.276	0.766
Smoking (yes/no)	64/12	70/19	59/11	0.362	0.990	0.367
AFP (≤400/>400, umol/L)	37/39	39/50	30/40	0.532	0.480	0.903
HbsAg (positive/negative)	69/7	81/8	64/6	0.961	0.892	0.927
TBIL (≤17.1/>17.1, umol/L)	58/18	69/20	49/21	0.854	0.389	0.281
Tumor diameter (cm)	8.50 ± 2.44	8.67 ± 2.02	8.58 ± 2.30	0.646	0.980	0.785
Tumor number (singly/multiply)	45/31	49/40	41/29	0.591	0.938	0.657

XCHD: Xiao-Chaihu-Decoction; NM: naturopathic medicine. XCHD group, application of the addition and subtraction theory of TCM using Xiao-Chaihu-Decoction; NM group, receiving naturopathic medicine therapy; combined group, Xiao-Chaihu-Decoction combined with naturopathic medicine therapy. KPS, Karnofsky performance score; AFP, alpha fetal protein; TBIL, total bilirubin. *P* < 0.05 indicated a statistically significance, indexes without statistical difference between groups by *χ* ^2^ test also showed none apparent statistical difference among groups by the one-way analysis of variance (ANOVA) test.

**Table 2:** Tumor responses associated with different therapies evaluated by new Response Evaluation Criteria in Solid Tumors (RECSIT).

Group	Cases (*N*)	CR (*N*)	PR (*N*)	SD (*N*)	PD (*N*)	ORR = CR + PR (*N*, %)	OSD = CR + PR + SD (*N*, %)
XCHD group	54	0	19	18	22	19 (35.2%)	37 (68.5%)
NM group	56	0	22	16	18	22 (39.3%)	38 (67.9%)
Combined group	42	2	18	11	9	20 (47.6%)	31 (73.8%)

XCHD: Xiao-Chaihu-Decoction; NM: naturopathic medicine; CR: complete remission; PR: partial remission; SD: stable duration; PD: progression disease. XCHD group, application of the addition and subtraction theory of TCM using Xiao-Chaihu-Decoction; NM group, receiving naturopathic medicine therapy; combined group, Xiao-Chaihu-Decoction combined with naturopathic medicine therapy.

**Table 3:** Body weight associated with different therapies evaluation among groups.

Group	Cases (*N*)	Increased/Stable (*N*, %)	Losing ≤ 5% (*N*, %)	Losing > 5% (*N*, %)
XCHD group	76	32 (42.1%)	23 (30.3%)	21 (27.6%)
NM group	89	43 (48.3%)	16 (18.0%)	30 (33.7%)
Combined group	70	49 (70.0%)	9 (12.9%)	12 (17.1%)

XCHD: Xiao-Chaihu-Decoction; NM: naturopathic medicine. XCHD group, application of the addition and subtraction theory of TCM using Xiao-Chaihu-Decoction; NM group, receiving naturopathic medicine therapy; combined group, Xiao-Chaihu-Decoction combined with naturopathic medicine therapy.
